# Phase II study to evaluate combining gemcitabine with flutamide in advanced pancreatic cancer patients

**DOI:** 10.1038/sj.bjc.6600523

**Published:** 2002-09-23

**Authors:** P Corrie, A Mayer, J Shaw, S D'Ath, S Blagden, C Blesing, P Price, N Warner

**Affiliations:** Oncology Centre, Addenbrooke's Hospital, Cambridge CB2 2QQ, UK; Department of Clinical Oncology, Churchill Hospital, Oxford OX3 7LJ, UK; Department of Clinical Oncology, Hammersmith Hospital, London W12 0HS, UK

**Keywords:** pancreatic cancer, gemcitabine, flutamide

## Abstract

A phase II study was undertaken to determine the safety of combining flutamide with gemcitabine, with response rate being the primary end point. Twenty-seven patients with histologically proven, previously untreated, unresectable pancreatic adenocarcinoma received gemcitabine, 1 g m^−2^ intravenously on days 1, 8 and 15 of a 28 day cycle, and flutamide 250 mg given orally three times daily. Treatment was halted if there was unacceptable toxicity, or evidence of disease progression. Toxicity was documented every cycle. Tumour assessment was undertaken after cycles 2 and 4, and thereafter at least every additional four cycles. One hundred and seventeen cycles of treatment were administered, median four cycles per patient (range 1–18). Gemcitabine combined with flutamide was well tolerated, with most toxicities being recorded as grade 1 or 2 and only nine treatment cycles associated with grade 3 toxicity. The most frequent toxicity was myelosuppression. One case of transient jaundice was recorded. The commonest symptomatic toxicity was nausea and vomiting. The response rate was 15% (four partial responses), median survival 6 months and 22% of patients were alive at 1 year. These results suggest antitumour activity of the combination therapy to be equivalent to single agent gemcitabine.

*British Journal of Cancer* (2002) **87**, 716–719. doi:10.1038/sj.bjc.6600523 www.bjcancer.com

© 2002 Cancer Research UK

## 

Pancreatic cancer is the fifth most common cause of adult death from malignancy, being responsible for nearly 5% of all cancer deaths. Unfortunately, to date, over 80% of patients at diagnosis present with advanced disease not amenable to surgery and their median life expectancy is around 4 months (Kelly and Benjamin, 1995). Pancreatic adenocarcinoma is both chemo- and radioresistant, with few single agent chemotherapy drugs achieving a response rate above 10%. In the pivotal prospective multicentre randomised trial comparing the deoxycitidine analogue, gemcitabine (2′,2′-difluorodeoxycitidine, Gemzar; Eli Lilly and Co. Ltd), 1 g m^−2^ 30 min infusion given weekly, with 5-fluorouracil (5FU), 600 mg m^−2^ 30 min infusion given weekly, in previously untreated pancreatic cancer patients (Burris *et al*, 1997), response rate, median survival and 1 year survival were 5.4%, 5.65 months and 18% for the gemcitabine arm, compared with 0%, 4.41 months and 2% for the 5FU arm. More clinically meaningful effects on disease-related symptoms (pain control, performance status and weight gain) were seen in gemcitabine-treated patients, with 24% achieving a clinical benefit response compared with only 5% of 5FU-treated patients. Despite these limited patient benefits, gemcitabine is now internationally accepted as standard systemic chemotherapy for such patients with unresectable disease. However, there is a clear need to identify more effective treatments.

Recently, a small single centre randomised placebo-controlled trial of the oral antiandrogen, flutamide, in unresectable pancreatic cancer was published (Greenway, 1998). Median survival was 8 months in the flutamide arm and 4 months in the placebo arm. Excluding patients who progressed within 6 weeks of treatment, these figures were 12 and 5 months respectively. These results support some preclinical data suggesting that testosterone may be a growth factor for pancreatic cancer. Androgen receptors have been demonstrated in human pancreatic cancer tissue (Corbishley *et al*, 1986), together with the steroid synthetic enzymes, aromatase and 5αreductase (Iqbal *et al*, 1983). In addition, patients with pancreatic cancer appear to have low serum testosterone concentrations (Greenway *et al*, 1983). Finally, testosterone has been shown to promote the growth of human pancreatic adenocarcinoma xenografts grown in nude mice, while an antiandrogen inhibited this effect (Greenway *et al*, 1982). Greenway's trial of 49 patients has been criticised for its small size and histological evidence of the disease was obtained in only 35% of cases. Even so, the suggestion that androgen receptor blockade may significantly improve survival of pancreatic cancer patients warrants further testing. Furthermore, the potential to combine flutamide with gemcitabine is attractive, since each single agent has a side effect profile acceptable for pancreatic cancer patients who are frequently elderly, frail and plagued with disease-related symptoms. We therefore undertook a phase II study to evaluate the combination regimen of gemcitabine and flutamide for safety, tolerability and efficacy.

## PATIENTS AND METHODS

### Patient eligibility

The eligibility criteria were histological or cytological diagnosis of previously untreated, unresectable (locally advanced or metastatic) pancreatic adenocarcinoma. Patients with previously resected disease who had received adjuvant therapy could be entered if relapse occurred more than 6 months from the date of completing previous therapy. Measurable disease was required, defined as evidence of any tumour mass which could be measured bidimensionally either clinically or radiologically. Baseline CT scans were performed within 4 weeks prior to commencing treatment. Patients had to be at least 18 years of age, with Eastern Cooperative Oncology Group performance status (PS) ⩽2. Laboratory parameters were as follows: Hb ⩾10 g dl^−1^, platelets⩾100 000 mm^−3^, ANC ⩾1500 mm^3^; bilirubin <1.3×ULN, Alk phos <2.5×ULN, transaminases <2.5×ULN; serum Creatinine <1.5×ULN. Patients could be entered beyond 7 days after major surgery or 3 days after laparoscopy. All patients gave written informed consent and the study was approved by the local research ethics committees of the three participating UK centres: Addenbrooke's Oncology Centre, Cambridge, the Churchill Hospital, Oxford, and the Hammersmith Hospital, London.

### Systemic therapy

Gemcitabine was administered as a 30 min infusion, 1 g m^−2^ weekly on days 1, 8 and 15, for 3 consecutive weeks, on a 4 week cycle. Flutamide was commenced on day 1 and taken orally 250 mg three times daily. Toxicity was assessed using the National Cancer Institute common toxicity criteria. Gemcitabine was halted if grade 3 or more nonhepatic toxicity occurred, and recommenced on recovery at 25% reduced dose. Treatment was halted for grade 2 or more liver toxicity. On recovery to grade ⩽1, gemcitabine was restarted at full dose, but flutamide was reduced to 250 mg twice daily in the case of grade 2 liver toxicity and omitted altogether in the case of grade 3 liver toxicity.

The initial study treatment period was defined as four cycles in the first instance. Patients completing cycle 4 with stable or responding disease and without significant side effects were allowed to continue treatment if they wished. Treatment was discontinued if there was unacceptable toxicity, evidence of disease progression, at patient request, or if considered appropriate for any other reason by the patient's doctor.

### Study parameters

Physical examinations, PS, weight, blood count, renal and liver function tests were measured at baseline and on days 1, 8 and 15 of every 4 week cycle. Serum CA19.9 was measured at baseline and if raised, was repeated every 4 weeks. Baseline serum testosterone was also measured. Tumour assessment for measurable disease was performed within 4 weeks of commencing treatment. This was repeated after cycle 2 and 4 and thereafter, at least every four cycles. An initial chest X-ray was performed and repeated when the patient came off study.

Standard WHO criteria were used to assess tumour response. Complete response (CR) was defined as complete disappearance of all known disease determined by two observations not less than 4 weeks apart. Partial response (PR) was defined as a decrease of 50% or more in the sum of the products of the two maximum perpendicular diameters of assessable disease for at least 4 weeks, with no appearance of new lesions or progression of any lesion. Stable disease (SD) was defined as a less than 50% decrease or a less than 25% increase in the sum of the products of the two maximum perpendicular diameters of assessable disease. Progressive disease (PD) was defined as a 25% or more increase in the sum of the products of the two maximum perpendicular diameters of assessable disease or the development of any new lesions.

### Statistical considerations

Using the method described by Gehan (1961), an initial cohort of 14 patients were recruited. Further patients were recruited so that the standard error of the observed response rate would be less than or equal to 0.01. This ensured that if the treatment was active in 20% or more patients, the chance of erroneously rejecting it after the first 14 patients was 0.044. Any patient failing to be assessed for tumour response after cycle 2 required an additional patient to be recruited to the study. The time to treatment failure was defined as the time from start of treatment until disease progression was first detected or the patient went off study due to clinical deterioration in the absence of tumour measurements. Overall survival was measured from the start of treatment until death from any cause.

## RESULTS

### Patient characteristics

From March 1999 to July 2000, 27 patients were recruited at three UK centres. Their characteristics are listed in Table 1

**Table 1:**
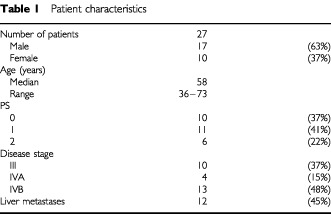
Patient characteristics

. All patients had histological confirmation of pancreatic adenocarcinoma. Twenty-four patients presented with locally advanced disease or metastatic tumours. Three patients had recurrence after primary resection, of whom two had received adjuvant chemoradiotherapy as part of the ESPAC 1 trial (Neoptolemos *et al*, 2001). At the time of censorship (1st January 2002), two patients remained alive at 95 and 134 weeks.

### Objective tumour responses

In the first cohort of 14 patients, four objective responses were recorded: three radiological partial responses (PRs) and one clinical PR. A further 13 patients were recruited, of whom three progressed within 6 weeks of treatment and 10 were assessable for response on completion of cycle 2. For the whole study population (*n*=27), the objective response rate was 15% (4 PRs). Of the remaining patients, 13 (48%) had stable disease of duration ranging between 14 and 76.3 weeks, with median 35.3 weeks. Ten (37%) patients had progressive disease documented at the first assessment.

The objective PRs occurred in two patients with locally advanced and two patients with disseminated disease, involving the liver in one case and left supraclavicular and axillary lymphadenopathy in the other. Responses were documented after cycle 2 in three cases and cycle 4 in one case. These responses were sustained for 6, 8, 27 and 16 weeks, respectively.

Serum testosterone measurements were recorded for only 60% of the study population. In 14 cases, testosterone was within the normal range and in only two cases was it below the lower limit of normal. There was no correlation of serum testosterone with disease stage or treatment outcome.

In this study, formal quality of life assessment was not undertaken, but patients were weighed and their PS determined prior to commencing each cycle of therapy. Response to treatment did not have any benefit to patients in terms of gain or maintenance of weight. However, patients who responded or whose disease remained stable maintained their PS for a statistically significantly longer period than those who progressed (*P*=0.046, paired student's *t*-test).

### Survival

The median time to disease progression for all 27 patients entered into this study was 18.1 weeks (range 1 to 76 weeks). To date, two patients remain alive and the median survival was 24.5 weeks (range 4 to 134 weeks), with 22% patients alive at 1 year.

### Toxicity

All patients were evaluated for toxicity. The median number of cycles of combination therapy received was 4, with range 1–18. Toxicity documented every cycle was generally mild (Table 2

**Table 2:**
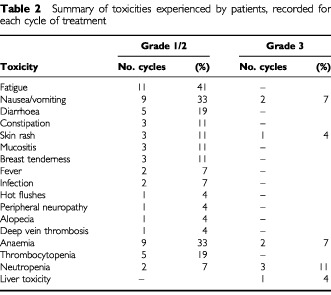
Summary of toxicities experienced by patients, recorded for each cycle of treatment

). However, haematological grade 3 toxicity was documented on five occasions, comprising neutropenia in three cases and anaemia in two cases. Thrombocytopenia was only ever documented as grade 1 or 2 and occurred on five occasions. One case of grade 3 liver toxicity (jaundice) occurred, which appeared to resolve on stopping flutamide. Three cases of symptomatic grade 3 toxicity were documented, comprising nausea and vomiting in two cases and skin rash in one case. The most frequently documented symptomatic grade 1/2 toxicities were fatigue (11 cases), nausea and vomiting (nine cases) and diarrhoea (five cases).

Sixteen patients had no modification of their treatment doses while on study. Modifications were made because of toxicity attributable to gemcitabine in eight patients and to flutamide in three patients. Reasons given for gemcitabine modification were haematological toxicity or severe skin rash, requiring three patients to stop gemcitabine treatment entirely. Flutamide was modified due to hot flushes or nausea, and in one patient who became jaundiced after two cycles of treatment, flutamide was omitted. Three male patients reported breast tenderness which was not considered severe enough to require treatment modification. No treatment related deaths were recorded.

## DISCUSSION

Pancreatic adenocarcinoma is known to be highly resistant to systemic therapy. The most effective single agent proven in randomised trials for treating advanced disease is the nucleoside antimetabolite, gemcitabine, but even with this treatment, benefits to patients are very limited. This trial was designed to evaluate the novel combination of gemcitabine and the antiandrogen, flutamide. On a practical basis, a well tolerated hormonal therapy would seem an attractive option in this group of highly symptomatic patients. However, flutamide can induce liver damage, as evidenced by transaminitis, cholestatic jaundice, liver necrosis, encephalopathy and, rarely, liver failure leading to death. Since many patients with pancreatic cancer have disease-related liver dysfunction, the concern was raised that patients entered into this study might be put at greater risk of flutamide-induced liver damage. In the randomised placebo-controlled trial of flutamide in advanced pancreatic cancer patients, flutamide was administered at a standard dose of 250 mg three times daily and no cases of liver toxicity were reported. Furthermore, in our own institution, a group of 26 patients with radiological evidence of advanced pancreatic cancer but without histological or cytological confirmation of disease patients were offered treatment with single agent flutamide. In this group, no liver-related side effects were reported (Shaw *et al*, 2000). The results of this study demonstrate that gemcitabine can be safely combined with flutamide in patients with advanced pancreatic adenocarcinoma. Few grade 3 and no grade 4, life threatening, toxicities were recorded. In a single patient who became jaundiced shortly after starting combination treatment, omission of flutamide led to resolution of liver blood tests and the patient was able to continue treatment with gemcitabine alone. 60% of patients were able to tolerate full doses of treatment without significant toxicity. Of those whose treatment was modified, the majority required dose reduction of gemcitabine for reasons predicted by the known side effect profile of this drug. There was no indication that combination therapy potentiated risk of toxicity from either drug.

The response rate associated with this treatment combination was 15%. This and the median survival of 6 months equate well with other phase II study data becoming available for gemcitabine-based combination regimens (Louvet *et al*, 2002), although many of these cytotoxic regimens are associated with more drug-related toxicity than described in this study.

In conclusion, it appears that gemcitabine may be safely combined with flutamide. The combination regimen appears to be at least as effective as, although probably not superior to single agent gemcitabine. Although testosterone was not measured in all study patients, the data does not support a previous report suggesting that all pancreatic cancer patients have low serum testosterone concentrations (Greenway *et al*, 1983). Further studies would be required to identify an antitumour effect of flutamide before further clinical trials in pancreatic cancer could be justified.
